# Prediction of Suicidal Behaviors in the Middle-aged Population: Machine Learning Analyses of UK Biobank

**DOI:** 10.2196/43419

**Published:** 2023-02-20

**Authors:** Junren Wang, Jiajun Qiu, Ting Zhu, Yu Zeng, Huazhen Yang, Yanan Shang, Jin Yin, Yajing Sun, Yuanyuan Qu, Unnur A Valdimarsdóttir, Huan Song

**Affiliations:** 1 West China Biomedical Big Data Center West China Hospital Sichuan University Chengdu China; 2 Med-X Center for Informatics Sichuan University Chengdu China; 3 Center of Public Health Sciences Faculty of Medicine University of Iceland Reykjavík Iceland; 4 Institute of Environmental Medicine, Karolinska Institutet Stockholm Sweden; 5 Department of Epidemiology Harvard T H Chan School of Public Health Harvard University Boston, MA United States

**Keywords:** suicide, suicidal behaviors, risk prediction, machine learning approach, genetic susceptibility, machine learning, behavior, data, model, sex, risk, cost-effective

## Abstract

**Background:**

Suicidal behaviors, including suicide deaths and attempts, are major public health concerns. However, previous suicide models required a huge amount of input features, resulting in limited applicability in clinical practice.

**Objective:**

We aimed to construct applicable models (ie, with limited features) for short- and long-term suicidal behavior prediction. We further validated these models among individuals with different genetic risks of suicide.

**Methods:**

Based on the prospective cohort of UK Biobank, we included 223 (0.06%) eligible cases of suicide attempts or deaths, according to hospital inpatient or death register data within 1 year from baseline and randomly selected 4460 (1.18%) controls (1:20) without such records. We similarly identified 833 (0.22%) cases of suicidal behaviors 1 to 6 years from baseline and 16,660 (4.42%) corresponding controls. Based on 143 input features, mainly including sociodemographic, environmental, and psychosocial factors; medical history; and polygenic risk scores (PRS) for suicidality, we applied a bagged balanced light gradient-boosting machine (LightGBM) with stratified 10-fold cross-validation and grid-search to construct the full prediction models for suicide attempts or deaths within 1 year or between 1 and 6 years. The Shapley Additive Explanations (SHAP) approach was used to quantify the importance of input features, and the top 20 features with the highest SHAP values were selected to train the applicable models. The external validity of the established models was assessed among 50,310 individuals who participated in UK Biobank repeated assessments both overall and by the level of PRS for suicidality.

**Results:**

Individuals with suicidal behaviors were on average 56 years old, with equal sex distribution. The application of these full models in the external validation data set demonstrated good model performance, with the area under the receiver operating characteristic (AUROC) curves of 0.919 and 0.892 within 1 year and between 1 and 6 years, respectively. Importantly, the applicable models with the top 20 most important features showed comparable external-validated performance (AUROC curves of 0.901 and 0.885) as the full models, based on which we found that individuals in the top quintile of predicted risk accounted for 91.7% (n=11) and 80.7% (n=25) of all suicidality cases within 1 year and during 1 to 6 years, respectively. We further obtained comparable prediction accuracy when applying these models to subpopulations with different genetic susceptibilities to suicidality. For example, for the 1-year risk prediction, the AUROC curves were 0.907 and 0.885 for the high (>2nd tertile of PRS) and low (<1st) genetic susceptibilities groups, respectively.

**Conclusions:**

We established applicable machine learning–based models for predicting both the short- and long-term risk of suicidality with high accuracy across populations of varying genetic risk for suicide, highlighting a cost-effective method of identifying individuals with a high risk of suicidality.

## Introduction

According to the estimation of the Global Burden of Disease study, approximately 800,000 people die by suicide every year [1], which translates to the astonishing number of 1 person dying by suicide every 40 seconds. In the United Kingdom, there were 5691 deaths by suicide registered in England and Wales in 2019, which corresponded to an age-standardized rate of 11 deaths per 100,000 people [2]. Importantly, behind the number of suicidal deaths, there is a much higher incidence of suicide attempts requiring further research. From 2000 to 2010, a prospective study using data from 5 emergency departments in the United Kingdom identified 38,415 individuals who presented at an emergency department following a suicide attempt [3], among which only 261 (0.7%) died. This finding implies that the population targeted for suicide prevention, such as timely psychological support, is considerably larger. However, only 28% of people who attempt suicide in the United Kingdom have previously received psychiatric services [4]. Therefore, it is urgent to improve the identification of individuals at high risk for suicidality to improve suicide prevention.

The previous research suggests that the mechanisms of suicidality are complex and multifactorial [5], likely involving interactions between genetic, psychological (including traumatic experiences), and socioeconomic or other environmental factors [6,7]. This report might explain the suboptimal accuracy of suicidality prediction based on traditional statistical models, for example, with the area under the receiver operating characteristic (AUROC) curve reported to be 0.58 in a meta-analysis of 367 studies, which was only slightly better than a prediction of chance [8].

Alternatively, as tools that can deal with multidimensional data, artificial intelligence techniques (including machine learning) that have been widely used to uncover predictions of multiple diseases [9-11] might have the potential to improve the prediction of suicidality. Indeed, based on data from electronic medical records and mental health questionnaires, as well as sociodemographic factors, researchers have constructed machine learning models that obtained good performance (AUROC=0.590-0.930) for suicidality prediction in the high-risk population [12]. Likewise, more recent efforts to predict suicide attempts or deaths in the general population using this approach have yielded promising results, showing AUROC curves of 0.80 and 0.88 among men and women, respectively, in a Danish population and an AUROC curve of 0.857 among participants in the National Alcohol Epidemiological Survey in the United States [13,14]. However, prior studies did not consider several important factors, such as genetic background [7] and lifestyle factors (eg, diet, physical activity, and sleep) [15,16]. In addition, all these existing models require many input variables (2554 and 2978 inputted features for the Danish and US study, respectively), which have limited implications for daily practice.

Taking advantage of enriched information about suicidality and environmental factors, as well as the available individual-level genotyping data in UK Biobank, we aimed to construct applicable models using a machine learning approach (ie, with limited features) to predict suicidal behavior over both the short and long term. To test the robustness of our models, we validate them among individuals with different genetic risks of suicide.

## Methods

### Data Source

A prospective UK Biobank cohort recruited 502,507 participants aged 40 to 69 years across the United Kingdom between 2006 and 2010 [17], which coincides with a high-risk age group of suicide among men and women [18]. At recruitment, all participants filled out questionnaires covering information on sociodemographic, lifestyle, and health–related factors, with a physical examination and collection of biological samples performed during the initial assessment. After recruitment, a proportion was invited several years later to repeat the assessment. In that study, 20,334 participants received a first repeated assessment in 2012 and 2013 and 51,131 received a second repeated assessment visit in 2014.

To track health-related outcomes, UK Biobank data have been linked periodically to multiple national registries with the participants’ consent [17]. The inpatient hospital data were obtained through linked hospital records in England, Scotland, and Wales, which were mapped from the Hospital Episode Statistics in England, the Scottish Morbidity Record, and the Patient Episode Database in Wales [19]. Primary care data were obtained from multiple data suppliers, including the Phoenix Partnership and Egton Medical Information Systems, which cover approximately 45% of UK Biobank participants [20]. The mortality data were obtained from national death registers, such as the National Health Services (NHS) Digital Registry and the NHS Central Registry [21].

**Figure 1:**
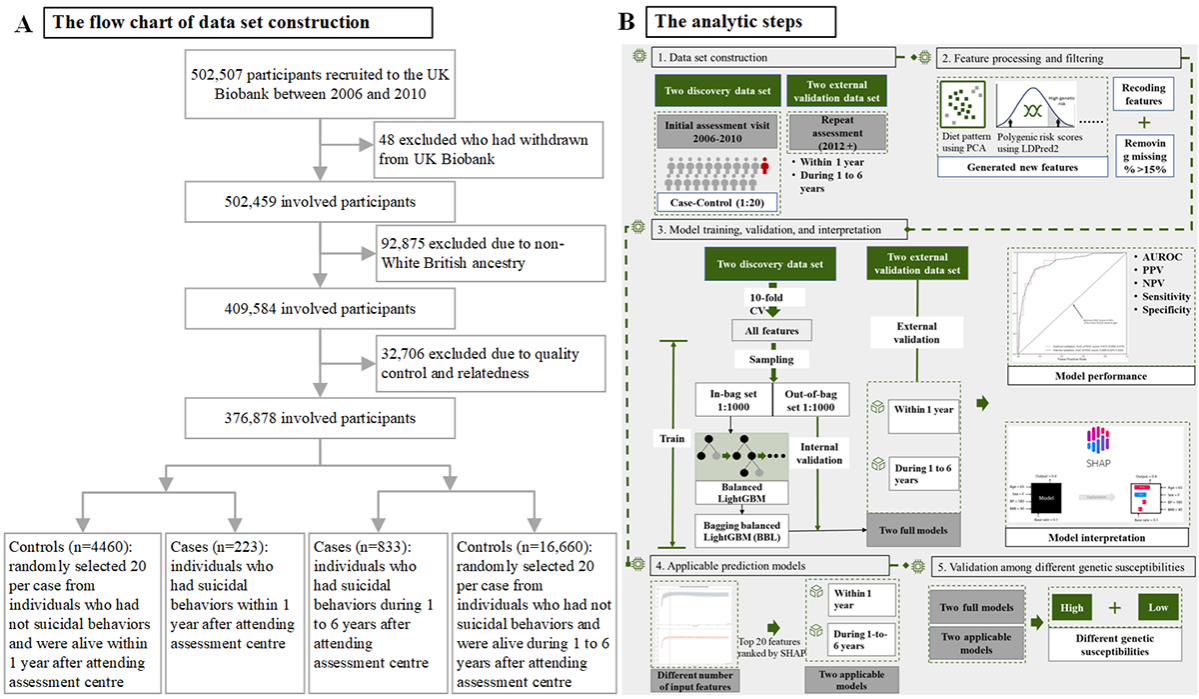
Flowchart of the study. AUROC: area under the receiver operator curve; CV: corss validation; LightGBM: light gradient-boosting machine; NPV: negative predictive value; PCA: principal component analysis; PPV: positive predictive value.

In this study, among the 502,507 UK Biobank participants, we excluded 48 individuals who had withdrawn from the UK Biobank. To ensure the measurement of genetic susceptibility for suicidality, 376,878 individuals with White ancestry and eligible genotyping data were included in the analysis (Figure 1A). Specifically, the polygenic risk score (PRS) was used as an index of genetic susceptibility, which was generated based on the genome-wide association study (GWAS) summary statistics (ie, effect sizes and standard errors for the variants) from an independent sample of 50,264 Danish residents involving 6,024 cases with an incidence of suicide attempt and 44,240 controls [22]. In addition to removing individuals with nonhomogenous European ancestry, this GWAS study applied principal components of genetic ancestry to take into account the effect of population stratification. We computed the PRS using LDPred2, a method of PRS calculation based on a matrix of correlations between genetic variants, which is faster, more accurate, and more robust than the LDPred14 [23]. In a validation step, the calculated PRS showed a high consistency with the studied phenotype (ie, suicidal behaviors) in our study population, yielding a mean area under the curve of 0.550 and an odds ratio of 2.34 (95% CI 1.66-3.29) by a unit increase in the PRS. During the analysis, we defined the genetic risk levels of suicidality as low (<1st tertile of the PRS), moderate (1st-2nd tertile), and high (>2nd tertile).

### Ethics Approval

UK Biobank has full ethical approval from the NHS National Research Ethics Service (16/NW/0274), and informed consent was obtained before data collection from each participant. This study was also approved by the biomedical research ethics committee of West China Hospital (2019-1171).

### Ascertainment of Suicidal Behaviors

To expand the application of our models to suicide prevention, both suicide attempts and deaths identified during the study period were considered suicidal behaviors of interest, which is consistent with previous studies [24,25]. Specifically, death by suicide was defined as death with suicide as the underlying cause of death and documented by its correspondence to the International Classification of Diseases 9th revision (ICD-9) and 10th revision (ICD-10) codes (ie, ICD-10: X60-84 and Y10-34; ICD-9: E950-958) [24,25] in the death register. Suicide attempts were considered as hospital admissions with a diagnosis of intentional self-harm (ICD 10: X60-84 and ICD-9: E950-958) or self-harm of undetermined intent (ICD-10: Y10-34) [24,25]. With relatively stable age- and sex-standardized incidence rates, the absolute number of suicide attempts and deaths was high within the first year of enrollment and dropped gradually to half that number in 6th year (Figure S1 of Multimedia Appendix 1 [26-28]). Thus, the outcomes of interest were suicidal behaviors occurring within 1 year (ie, short term) and 1 to 6 years (ie, long term) after the recruitment. We considered individuals with suicide attempts before the recruitment as those having a history of suicide attempts.

### Data Set Construction

We constructed separate data sets for predicting suicidal behaviors within 1 year and 1 to 6 years. For the short-term risk prediction, we identified cases of suicide attempts or deaths at least 1 time within 1 year after recruitment (n=223). Controls (n=4460) were randomly selected (1:20 allocation ratio) from the remaining participants who were eligible, alive, and free of suicidal behaviors 1 year after the recruitment, resulting in a data set consisting of 4683 participants (Figure 1A). The same strategies were applied to constructing data sets for long-term (ie, 1 to 6 years) suicide risk prediction, yielding a full data set of 17,493 participants, with 833 (4.8%) and 16,660 (95.2%) cases and controls, respectively.

The 2 aforementioned data sets were then used as discovery data sets for model training and the assessment of internal validity. We additionally used a subsample comprising 50,310 participants of White ancestry from UK Biobank who participated in the repeat assessments. Among this subsample, there were 12 (0.02%) and 31 (0.06%) individuals who attempted or died by suicide within 1 year or during 1 to 6 years after their repeat measurements, respectively, as the validation data set for assessing external validity.

### Feature Processing and Filtering

Taking full advantage of the diversity of variables in UK Biobank, we generated a feature list involving multidimensional factors. Due to difficulties obtaining individual genetic data in the real world, we did not involve the PRS in the construction of the prediction models, but we subsequently validated the suicide prediction models with the subgroups of varying (ie, high and low) genetic susceptibility to suicidality to demonstrate their robustness. Information regarding sociodemographic, environmental, and psychosocial factors was derived from the data collected at recruitment using the touchscreen or face-to-face interview questionnaires. For categorical variables (eg, “In general, how would you rate your overall health?”), UK Biobank assigns negative values to categories denoting missingness (ie, −1 refers to “Prefer not to answer,” and −3 refers to “Do not know”). Therefore, we recorded those negative values as “NA.” Specifically, instead of directly using variables collected through a generic diet questionnaire, we identified dietary patterns based on the results of principal component analysis with varimax rotation (Figure S2 of Multimedia Appendix 1). They were referred to as the prudent, western, and open-sandwich patterns [26], yielding variables with top factor loadings in each component (Table S1 in Multimedia Appendix 1). Medical data included the physical examinations (eg, pulse rate, blood pressure, and grip strength of both hands) conducted during the initial medical center visit, and we calculated mean values when multiple records existed. Additionally, a history of psychiatric disorders was defined as any previous diagnosis of psychiatric disorders before baseline (ICD-10: any F), which was identified through self-reported, hospital inpatient, and primary care data. To consider the influence of somatic fitness, we generated time-varying (0 to 1 and 1 to 4 years before the recruitment) dichotomous variables for each subtype of severe somatic diseases [29]. For the analyses of the total study population, the level of genetic susceptibility to suicidality (low, moderate, or high) was also considered a candidate feature.

After excluding variables with over 15% of missing or irrelevant data (eg, device ID, seated boxing height, and hair color), we included a total of 143 features. The coding book of the included features is shown in Table S2 of Multimedia Appendix 1.

### Model Training and Validation

We constructed prediction models using all eligible features. The balanced bagging algorithm is proven to have good performance for classification models with class-imbalanced data [14]. Moreover, the light gradient-boosting machine (LightGBM) [30], as a gradient-boosting algorithm, has been widely applied in machine learning research due to its fast computational speed, high accuracy, and ability to handle missing values [11]. Therefore, considering the data imbalance and the existence of missing values, we used the balanced bagging LightGBM approach to achieve high classification accuracy and fast computation speed, which bagged 1000 balanced LightGBM classifiers (ie, using “class_weight” =“balanced”) after random downsampling [31]. We tuned the parameters by using stratified 10-fold cross-validation and grid-search, with the best combination of hyperparameters shown in the Methods section of Multimedia Appendix 1. Each of the 1000 balanced LightGBM classifiers randomly selected subsamples from the group of the minority class (ie, those who had suicidal behavior) and matched samples with the same size from the group of the majority class (ie, those who had no suicidal behavior) to construct case-control samples (ie, the in-bag set). The randomly selected case-control samples were applied to train balanced LightGBM classifiers, and the remaining sample, referred to as the out-of-bag (OOB) set, was used to estimate the prediction of the suicide risk score of the OOB set.

We defined the OOB set as the internal validation set. Specifically, we aggregated the predicted suicide risk scores of the OOB set from the 1000 balanced LightGBM classifiers to estimate the internal validated predicted error [32], and we regarded the models with the highest OOB AUROCs as optimal. Then, we computed the predicted suicide risk scores of the externally validated data sets from the repeated assessments for the optimal model. Due to the lack of agreement regarding which of the risk thresholds of classification provides the most sufficient clinical utility, we computed the AUROC [13,24], sensitivity, specificity, positive predictive value (PPV), and negative predictive value (NPV) at different suicide risk score thresholds.

### Model Explanation

Interpretations of the models were measured using the Shapley Additive Explanations (SHAP) approach, which quantifies the relationship of the input features with the outcome [33]. Specifically, we computed the contribution of all the features to the studied suicidal behaviors for each participant and assigned each feature an importance score (ie, a SHAP value) after considering its interactions with the remaining features. The absolute values of the average SHAP values were presented as a bar plot illustrating the relative importance of these input features for the models’ predictions at the population level. 

### Applicable Prediction Models

To facilitate the application of the prediction models, we conducted feature reduction by illustrating the changes in the prediction accuracy of the models with different numbers of input features (ie, those with top 10, 20, 50, and 100 SHAP values) [34,35]. As shown in Figure S3 of Multimedia Appendix 1, the models for predicting suicidal behaviors within 1 year and from 1 to 6 years both achieved overall good performance when the input feature dimension with the highest SHAP value was increased to 20, so we considered the models with 20 input features as the applicable prediction models which might facilitate the future implication.

### Model Validation Among Individuals With Different Genetic Susceptibilities

To illustrate the robustness of the suicide prediction models, we validated both full and applicable models in the whole population as well as subgroups of varying (ie, high and low) genetic susceptibility to suicidality by computing the OOB performance of these models.

We performed the data set construction and calculation of the PRS using R software version 3.6.1 (Lucent Technologies Co). The machine learning model development was achieved using Python software version 3.6 (Software Foundation), imbalanced-learn 0.9.0, and lightgbm version 3.2.1. We conducted the model interpretation analysis using SHAP version 0.38.1. We then analyzed the models’ performance and plot creation using scikit-learn version 1.0.2 and matplotlib version 3.3.2, respectively.

## Results

### Study Population Characteristics

The data sets for the prediction of suicidal behavior prediction within 1 year and for 1 to 6 years showed largely comparable characteristics at baseline (Table 1). We obtained similar ages, with mean ages of 56.75 (SD 8.03) and 56.65 (SD 7.99) years, respectively, and female-to-male sex distributions of 1:1.13 and 1:1.20, respectively. However, the characteristics of the validation sample for external validity (ie, individuals involved in the repeat assessments) were different from the discovery sample (ie, individuals recruited in the initial assessment visit), characterized by older age, more likely to have a history of psychiatric disorders, and lived in their own accommodation at time of data collection (Table 1).

**Table 1:** Basic characteristics of analytic samples for the construction of prediction models for 1 year and 1 to 6 years.

Characteristics			Discovery				External validation (n=50,310)
			Within 1 year (n=4683)		1 to 6 years (n=17,493)		
Age (years), mean (SD)			56.75 (8.03)		56.65 (7.99)		63.24 (7.49)
**Gender, n (%)**							
	Female	2480 (53)		9537 (54.5)		25,675 (51)	
	Male	2203 (47)		7956 (45.5)		24,635 (49)	
**History of psychiatric disorders, n (%)**							
	No	3760 (80.3)		14,371 (82.2)		39,226 (78)	
	Yes	923 (19.7)		3122 (17.8)		11,084 (22)	
**History of suicide attempt n (%)**							
	No	4574 (97.7)		17,270 (98.7)		50,108 (99.6)	
	Yes	109 (2.3)		223 (1.3)		202 (0.4)	
**Have you ever seen a psychiatrist for nerves, anxiety, tension, or depression? n (%)**							
	No	3999 (85.4)		15,188 (86.8)		45,372 (90.2)	
	Yes	660 (14.1)		2248 (12.9)		4556 (9.1)	
	Missing	24 (0.5)		57 (0.3)		382 (0.8)	
**Have you ever seen a general practitioner for nerves, anxiety, tension, or depression? n (%)**							
	No	2882 (61.5)		11,181 (63.9)		34,168 (67.9)	
	Yes	1768 (37.8)		6215 (35.5)		15,685 (31.2)	
	Missing	33 (0.7)		97 (0.6)		457 (0.9)	
**In the past, how often have you smoked tobacco? n (%)**							
	Smoked on most or all days	1212 (25.9)		4237 (24.2)		11,900 (23.7)	
	Smoked occasionally	573 (12.2)		2231 (12.8)		6061 (12)	
	Just tried once or twice	685 (14.6)		2634 (15.1)		7983 (15.9)	
	I have never smoked	1806 (38.6)		6897 (39.4)		22,789 (45.3)	
	Missing	407 (8.7)		1494 (8.5)		1577 (3.1)	
**Do you live in your own accommodation? n (%)**							
	No	2194 (46.9)		8136 (46.5)		11,670 (23.2)	
	Yes	2414 (51.5)		9155 (52.3)		38,114 (75.8)	
	Missing	75 (1.6)		202 (1.2)		526 (1)	
**Average annual total household income before tax^a^, n (%)**							
	Less than £18,000 (US $16,676)	945 (20.2)		3410 (19.5)		6502 (12.9)	
	£18,000 to £30,999 (US $16,676 to $28,718)	1045 (22.3)		3802 (21.7)		13,250 (26.3)	
	£31,000 to £51,999 (US $28,719 to $48,173)	1073 (22.9)		3958 (22.6)		13,577 (27)	
	£52,000 to £100,000 (US $48,174 to $92,642)	816 (17.4)		3113 (17.8)		9529 (18.9)	
	Greater than £100,000 (US $92,642)	194 (4.1)		797 (4.6)		2614 (5.2)	
	Missing	610 (13)		2413 (13.8)		4838 (9.6)	
Cases, n (%)			223 (47.62)		833 (47.62)		12 (0.02)^b^ and 31 (0.06)^c^

^a^Note that income data were collected between 2008 and 2010. Income was converted to US dollars according to the current exchange range.

^b^Refers to the number of cases of suicidal behaviors within 1 year.

^c^Refers to the number of cases of suicidal behaviors for 1 to 6 years.

### Prediction Models Involving All Features

**Figure 2:**
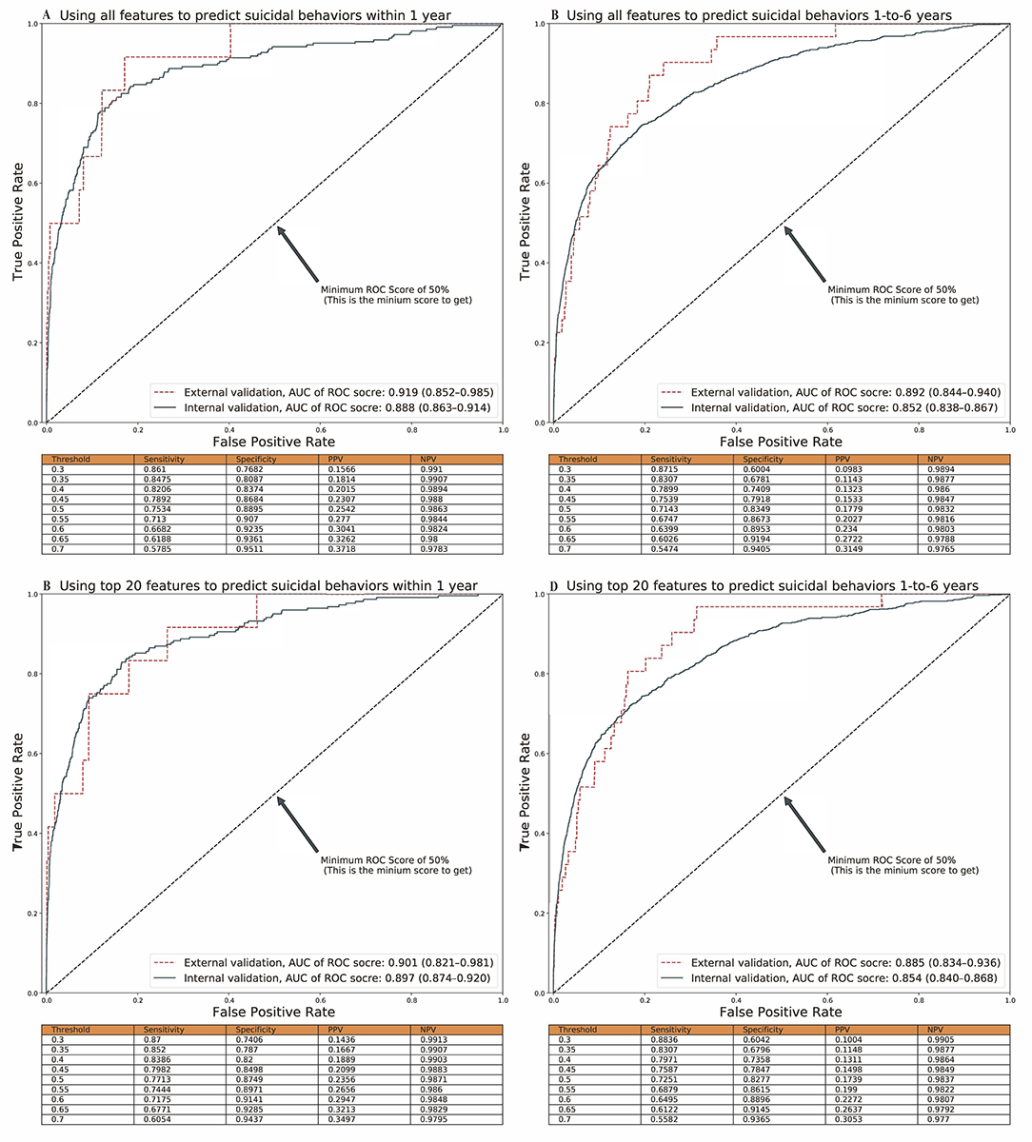
The performance of prediction models using all input features and top 20 features. The area under the receiver operating characteristic (AUROC) curve. The tables showed the internal validation performance (ie, sensitivity, specificity, positive predictive value [PPV], and negative predictive value [NPV]) of suicide prediction models at different classified thresholds.

The internal validated AUROC of the prediction models involving all features was 0.888 (95% CI 0.863-0.914) for the prediction of suicidal behaviors within 1 year and 0.852 (95% CI 0.838-0.867) for 1 to 6 years (Figure 2). Figure 2 shows values of sensitivity, specificity, and predictive indices over a series of risk thresholds. For instance, at the 0.70 risk threshold, the short- and long-term sensitivities were, respectively, 57.85% and 54.74%, the specificities were 95.11% and 94.05%, the PPVs were 37.18% and 31.49%, and the NPVs were 97.83% and 97.65%. Furthermore, the externally validated AUROC curves were 0.919 (95% CI 0.852-0.985) for the model predicting suicidal behaviors within 1 year and 0.892 (95% CI 0.844-0.940) for the model predicting suicidal behaviors between 1 and 6 years, indicating the robustness of the prediction models.

**Figure 3:**
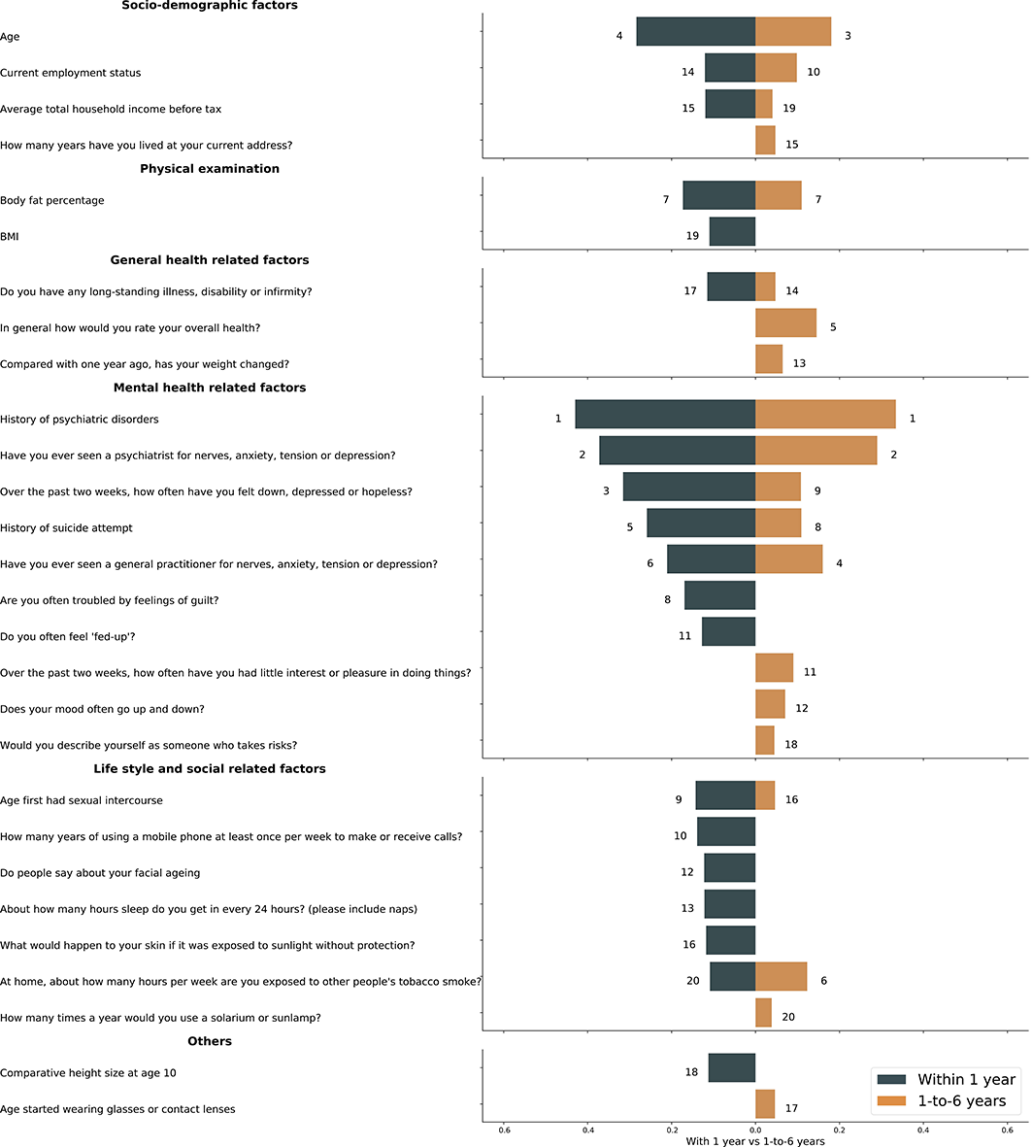
The comparison of top 20 features identified in suicide risk prediction full models for within 1 year and 1-to-6 years. The dark blue and yellow bar represent the relatively importance of these input features for the prediction, respectively. And the numbers next to the bars are corresponding to the ranking of top 20 features. The detailed information of the included features is shown in Table S2 in Multimedia Appendix 1.

Regarding the importance of features measured using SHAP values, similar to age, family income, and body fat percentage, mental health–related factors (eg, history of psychiatric disorders, history of suicide attempt, etc) were top ranked in both models within 1 year (7 among the top 20 features) and during 1 to 6 years (8 among the top 20 features). However, notable differences were observed with respect to some lifestyle and social factors (eg, “How many years of using a mobile phone at least once per week to make or receive calls?” and “Age you first had sexual intercourse”), as these seemed to have greater importance for only the prediction models within 1 year but not 1 to 6 years (Figure 3). In contrast, some general health–related factors (ie, “In general how would you rate your overall health?” and “Compared with 1 year ago, has your weight changed?”) seemed only important for the 1-to-6 years prediction model. Detailed information on the included features is shown in Table S2 of Multimedia Appendix 1.

### Prediction Models Involving the Top 20 Features

Figure S3 of Multimedia Appendix 1 displays the indices of model performance for the models involving different numbers of the top features (ie, top 20, 40, 60, and 100). Accordingly, the 2 models with the top 20 input features were considered optimal (Figure 2). The AUROC curves for their internal and external validations for the within 1-year suicide prediction were 0.897 (95% CI 0.874-0.920) and 0.901 (95% CI 0.821-0.981), respectively. For the 1-to-6 years prediction, the corresponding estimate was 0.854 (95% CI 0.840-0.868) and 0.885 (95% CI 0.834-0.936), respectively. Based on the applicable models, we found individuals in the top quintile of predicted risk accounting for 91.7% (n=11) and 80.7% (n=25) of all cases of suicide attempts or deaths within 1 year and during 1 to 6 years, respectively.

### Models for Individuals With Different Genetic Susceptibilities

Using both full and simplified prediction models, we obtained a comparable prediction accuracy for individuals with low and high genetic susceptibilities to suicidality (Figures S4 and S5 of Multimedia Appendix 1). For instance, for short-term risk prediction, the AUROC curves for models with the top 20 involved features were 0.907 and 0.885 for the high and low genetic susceptibility groups, respectively. The corresponding numbers for the long-term risk prediction were 0.869 and 0.822, respectively.

## Discussion

### Principal Findings

In this study on a community-based UK Biobank cohort of over 0.5 million UK residents aged 40 to 69 years (covering the age group with a high risk of suicide [18]), we established machine learning–based models to accurately predict both short- and long-term risks of suicide attempts and deaths (AUROC=0.892-0.919). Importantly, our applicable models achieved high predictive accuracy across populations with varying genetic susceptibility to suicide with a limited number (ie, 20) of phenotypic features that could be accessed easily through practice. Specifically, we found that individuals with the top 20% of predicted risks comprised over 80% of real cases of suicide attempts or deaths, suggesting that our approach may be a cost-effective way to identify high-risk middle-aged individuals who should be targeted for suicide prevention. In addition, besides some well-known suicide risk factors (ie, mental health–related conditions), these established models provide novel insights into factors driving suicidal behaviors, revealing that some lifestyle and social factors (eg, cell phone use frequency, etc) may be risk factors for suicidal behaviors in the short-term, while self-reported general health ratings are more important for the prediction of long-term suicidal risk.

In line with 2 previous studies focusing on machine learning–based suicide risk prediction in the general population using data from Danish health registers [13] and the National Alcohol Epidemiological Survey of the United States [14], our results identified mental health–related factors (ie, prior suicide attempt, history of psychiatric disorders, and past emotion) and sociodemographic factors (ie, age and family income) as top features for suicide risk prediction. However, benefiting from the enriched data in UK Biobank, particularly items related to neuroticism, lifestyle, social contacts, and self-rated general health, our prediction models achieved improved performance. In addition, the comparison of features that matter for short- versus longer-term suicide risk was not addressed in prior investigations. Similar efforts have been made in some specific populations (eg, patients receiving psychiatric [24] or other medical care [36] and soldiers [37]), though with only comparable predictive accuracy (ie, the AUROC curves ranged between 0.77 and 0.93) with more homogeneous clinical populations.

Consistent with our findings, neuroticism was reported as a risk factor for suicidal behaviors in a previous study, with plausible mechanisms of shared genetic components [38]. Likewise, severe somatic diseases, disabilities, or physical weakness have consistently been reported to be associated with higher suicide risk, which is possibly due to the chronic stress associated with these diagnoses and living with these diseases [7]. Previous efforts exploring the association between BMI and suicidality have led to inconsistent results [39], and the association between body fat and suicidality has remained largely unexplored. Nevertheless, our findings of the association between body fat percentage and suicidality gain support from a Mendelian randomization analysis, which revealed a causal link between a high percentage of body fat and depression [40].

Our attempts to construct separate models for the prediction of both short- and long-term suicide risks indicated that the models generally achieved better prediction accuracy for the more immediate period before the suicide attempt or death, which is in line with the findings of prior studies concerning time-varying suicide risk assessments [24,41]. While factors directly reflecting mental health impairment show consistent importance for both short- and long-term suicidal risk prediction, the significance of lifestyle and social factors (eg, the frequency of using a cell phone to make or receive calls) was mainly observed for short-term risk (ie, within 1 year), indicating the role of lower social support and social relations among individuals with suicide risk [7]. Additionally, our findings on the association between self-reported health ratings and long-term suicide risk are in line with the results of the Danish study, which also found that medical diagnoses and medications related to some somatic illnesses (eg, infection and respiratory diseases) measured 48 months before suicide were more important indicators of suicide risk than those measured 6 months earlier [13].

### Strengths and Weaknesses

The major merits of our study include the use of multidimensional data (including individual-level genotyping data) from a large community-based cohort of UK Biobank. The application of the machine learning approach, together with the use of SHAP values for feature interpretation, enabled us to identify the most informative variables that maximized the efficiency of the data for an accurate prediction of suicide risk. The imbalance in the sample sizes of the cases and the controls was mitigated by randomly downsampling and setting class weights for imbalanced classes in LightGBM during the training step [30,42]. Further, we improved the feasibility of our prediction models by using the feature reduction process, where accurate classification was achieved with only 20 features. Although no similar data from independent samples could be used for external validation, the validity of our models was demonstrated in a subgroup of UK Biobank participants who repeated surveys many years after the baseline measurement (showing different basic characteristics compared to the discovery data set), as well as the subpopulations stratified by their level of genetic susceptibility to suicidality.

A notable limitation of this study is the absence of data from emergency care departments, which were the main source for suicide case identification in previous studies [13,43]. Therefore, our study focused on suicidal behaviors resulting in hospitalization or death, and those with less severe consequences require further investigation. In addition, it is difficult to distinguish suicide attempts from nonsuicidal intentional self-harm based on ICD codes, as clinical diagnoses tended to be consequence oriented (ie, leading to life-threatening harm or not) or dependent on self-reported reasoning on intent. Moreover, such outcome ascertainment strategies have been demonstrated to suffer from poor sensitivity, resulting in a risk of underestimation of suicidal cases, as well as attenuated associations between studied exposures and suicidal outcomes [44]. Nevertheless, as this is the most feasible method to identify suicidal behavior, similar definitions and ascertainment of suicidal behaviors have been widely used in other large community- or population-based studies with a similar focus [24,25]. Furthermore, we only used the LightGBM as the base estimator for bagging, mainly due to its capability to handle missing values and achieve high discrimination accuracy [30]. It is possible that other machine learning approaches (eg, deep neural network), with some common methods of feature engineering (eg, standardization, one-hot encoding), might obtain better performance at the price of model interpretability. Finally, the UK Biobank study recruited only 5.5% of the invited individuals in the age range of 40 to 69 years, leading to a selection bias of the study population compared to the entirety of the population in the United Kingdom [45]. Consequently, the generalization of our findings to the total UK population and other populations cannot be made.

### Conclusions

In conclusion, based on a UK Biobank cohort, we established clinically applicable machine learning–based models for accurately predicting both short- and long-term risks of suicidal behaviors. The good performance of the models for subgroups with different genetic susceptibilities to suicidality highlights the possibility of applying these models to high-risk individual identification in the general middle-aged population, which may facilitate the development of cost-effective suicide prevention.

## Appendix

Multimedia Appendix 1 Supplementary methods, tables, and figures.

## Abbreviations

AUROC: area under the receiver operating characteristic
GWAS: genome-wide association study
ICD-9/ICD-10: International Classification of Diseases 9th revision/10th revision
LightGBM: light gradient-boosting machine
NHS: National Health Services
NPV: negative predictive value
OOB: out of bag
PPV: positive predictive value
PRS: polygenic risk score
SHAP: Shapley Additive Explanations

## Data Availability

Data from the UK Biobank are available to all researchers upon submitted application. All codes associated with the current submission are available and can be requested by contacting the corresponding authors.
