# Supplementary material for: Prediction of Suicidal Behaviors in the Middle-aged Population: Machine Learning Analyses of UK Biobank
Source: JMIR Public Health Surveill. 2023 Feb 20;9:e43419. doi: 10.2196/43419 (PMC9989910; doi:10.2196/43419)
Supplement: Multimedia Appendix 1 [file publichealth_v9i1e43419_app1.docx]

**SUPPLEMENT**

**METHODS**

**FIGURES**

**Figure S1** The absolute numbers and standardized incidence rates of suicide attempts or deaths from attending assessment --------------------------------------2

**Figure S2** The scree plot ------------------------------------------------------------------------2

**Figure S3** The ROC-AUC of models using different numbers of top feature to predict suicide behaviors ---------------------------------------------------------------------------------3

**Figure S4** The performance of prediction models using all input features (i.e., full models) among individuals with low and high genetic susceptibilities to suicidality --4

**Figure S5** The performance of applicable models, based on top 20 features identified by the full prediction models, among individuals with low and high genetic susceptibilities to suicidality -------------------------------------------------------------------5

**TABLES**

**Table S1** The loadings of PCA ----------------------------------------------------------------6

**Table S2** The coding Book ----------------------------------------------------------------7~10

**METHODS**

***Ascertainment of dietary patterns***

In the UK Biobank, all participants filled out a generic diet questionnaire, which was used to estimate the average consumption of fruits, vegetables (raw and cooked), fish (oily and non-oily), meat (processed, beef, lamb, pork), bread, cheese, cereal, and drinking water. We further imputed the missing values of continuous variables and ordinal categorical variables with the mean and median respectively. To identify dietary patterns, the consumption diet questionnaire was standardized and included in the principal component analysis (PCA) with varimax rotation [27, 28]. We determined the number of factors to be 3 through the eigenvalue’s scree plot (supplementary figure 2). Finally, we defined three diet patterns: prudent pattern, western pattern, and open-sandwich pattern [26], whose rotated factor loadings are shown in supplementary table 1.

***The best combination of hyperparameters***

The full and applicable prediction models, to predict both short- and long-term suicidal behaviors, was trained to optimize the AUC-ROC using stratified 10-fold cross-validation and grid-search. The best combination of hyperparameters were as follows. 1) long-term full prediction models: learning_rate = 0.1, max_depth = 25, num_leaves = 3, and reg_alpha = 1.0. 2) Short-term full prediction models: learning_rate = 0.3, max_depth = 25, num_leaves = 3, and reg_alpha = 1.0. 3) Long-term applicable prediction models: learning_rate = 0.1, max_depth = 25, num_leaves = 3, reg_alpha = 1.5. 4) Short -term applicable prediction models: learning_rate = 0.3, max_depth = 25, num_leaves = 3, and reg_alpha = 1.5.

| 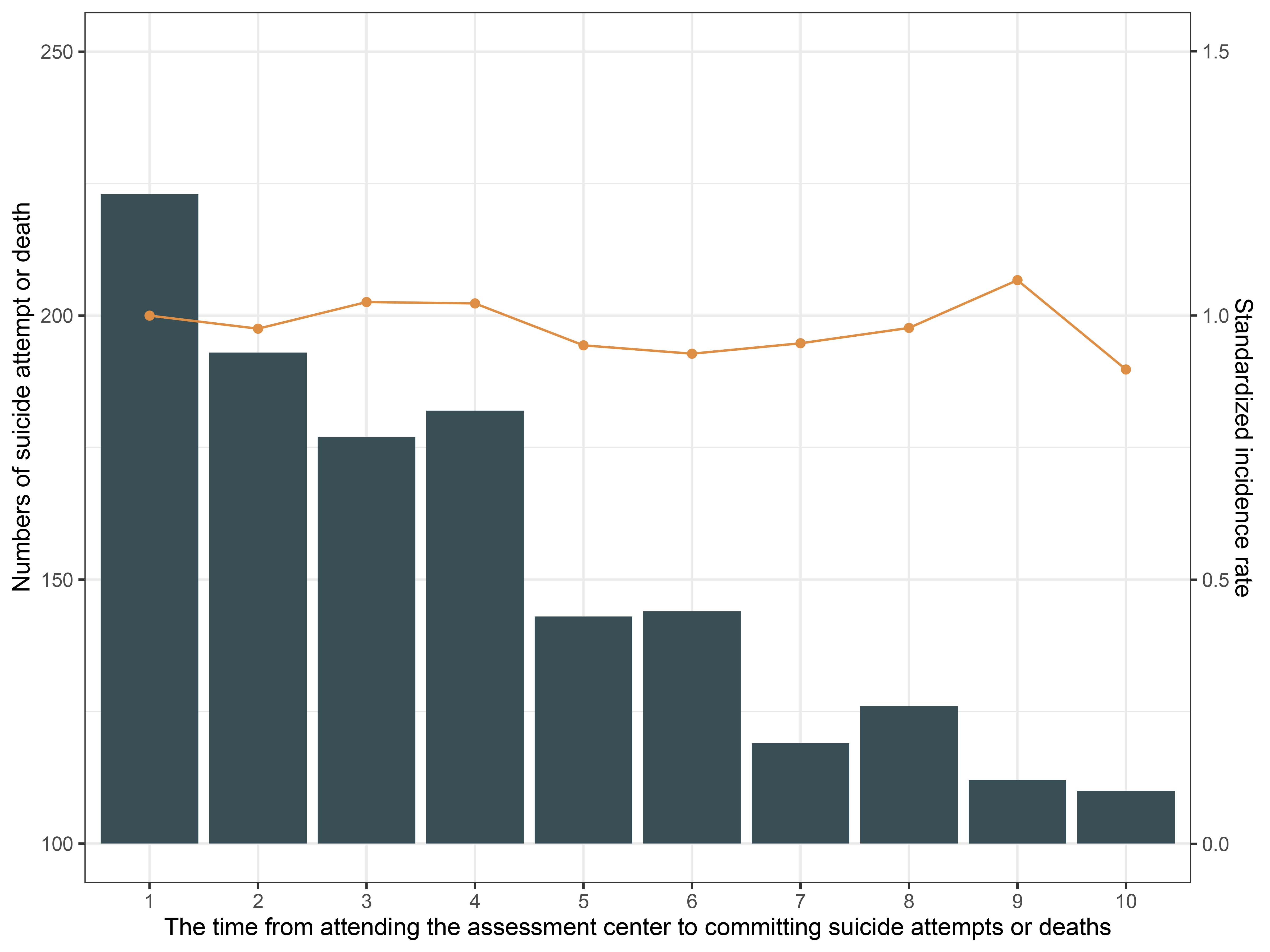 |
| --- |
| Figure S1 The absolute numbers and standardized incidence rates of suicide attempts or deaths from attending assessment  The first year from attending assessment center was used as the reference period. To control for aging of the study population, standardized incidence rates (i.e., adjusted by age and sex) were used to estimate the relative risk of suicide attempts or deaths, compared to the reference period. |

| **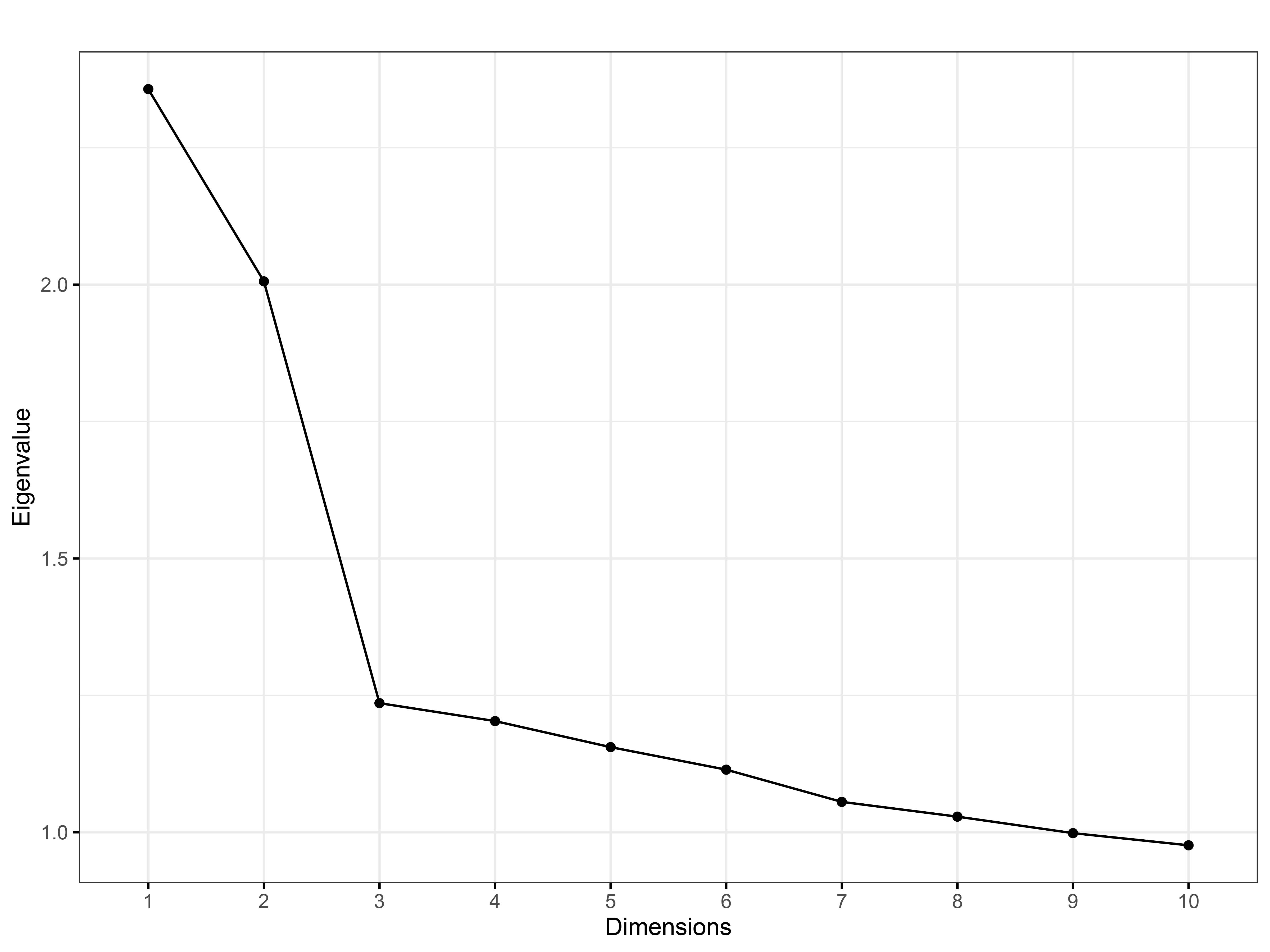** |
| --- |
| Figure S2 The scree plot |

| **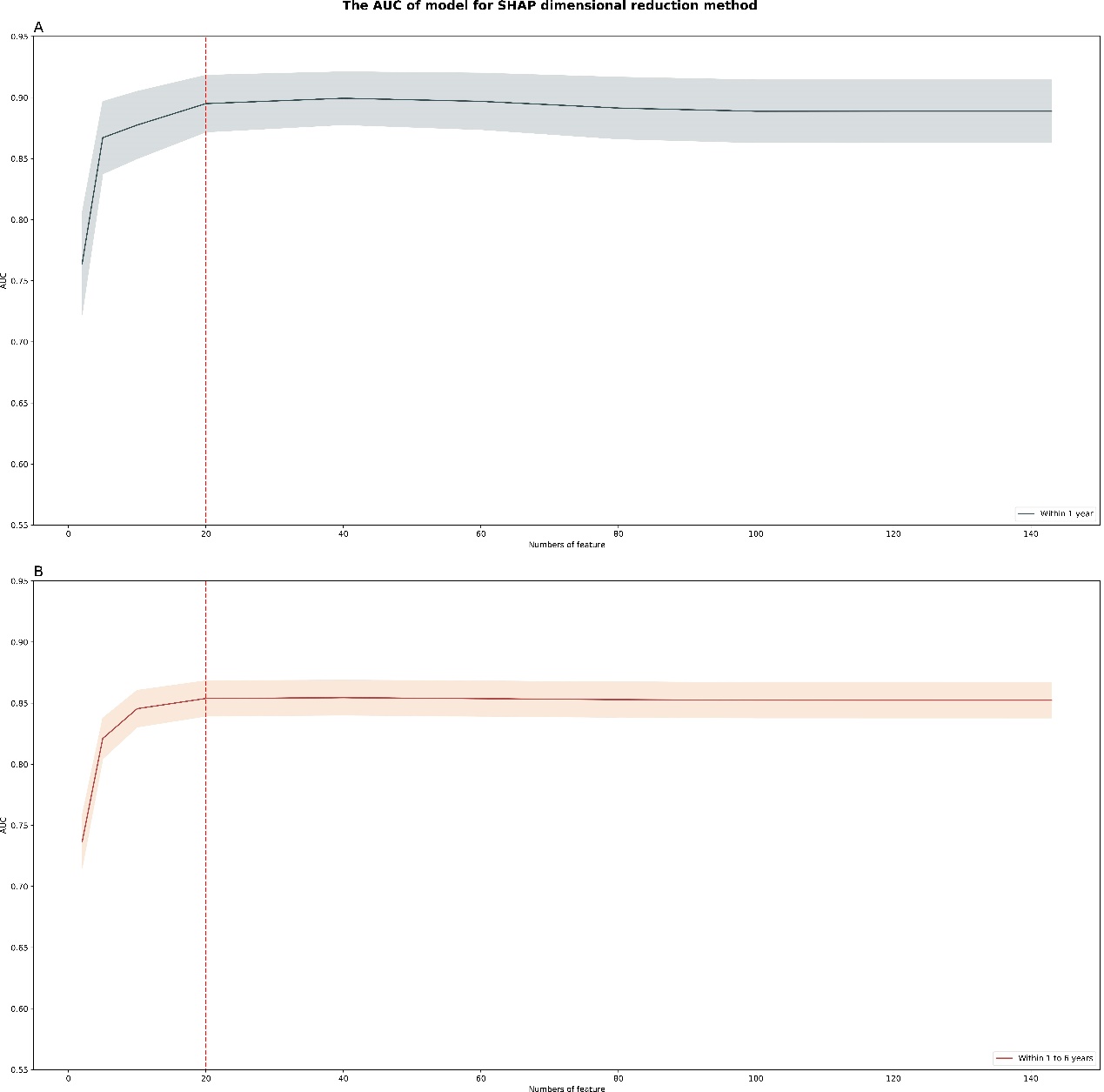** |
| --- |
| Figure S3 The AUC-ROC of models using different numbers of top feature to predict suicide behaviors  The area under the receiver operator curve (AUC-ROC).  Models to predict suicidal behaviors with 1 year and 1-to-6 years both achieved an overall good performance when the feature dimension was increased to 20 |

| **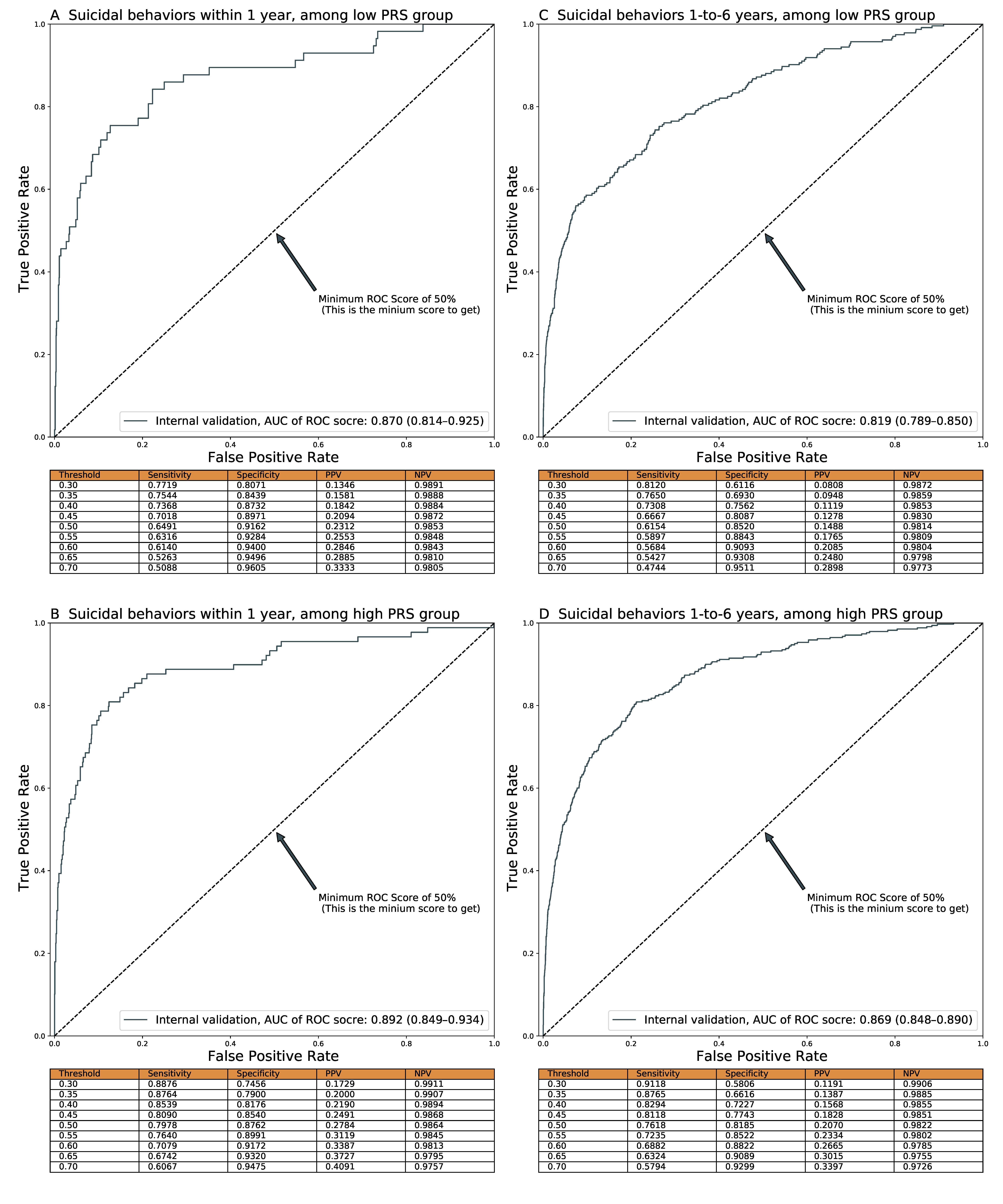** |
| --- |
| Figure S4 The performance of prediction models using all input features (i.e., full models) among individuals with low and high genetic susceptibilities to suicidality  The area under the receiver operator curve (AUC-ROC).  The above tables showed the internal validation performance (sensitivity, specificity, positive predictive value (PPV), and negative predictive value (NPV)) of suicide predictions models at different classified threshold. |

| **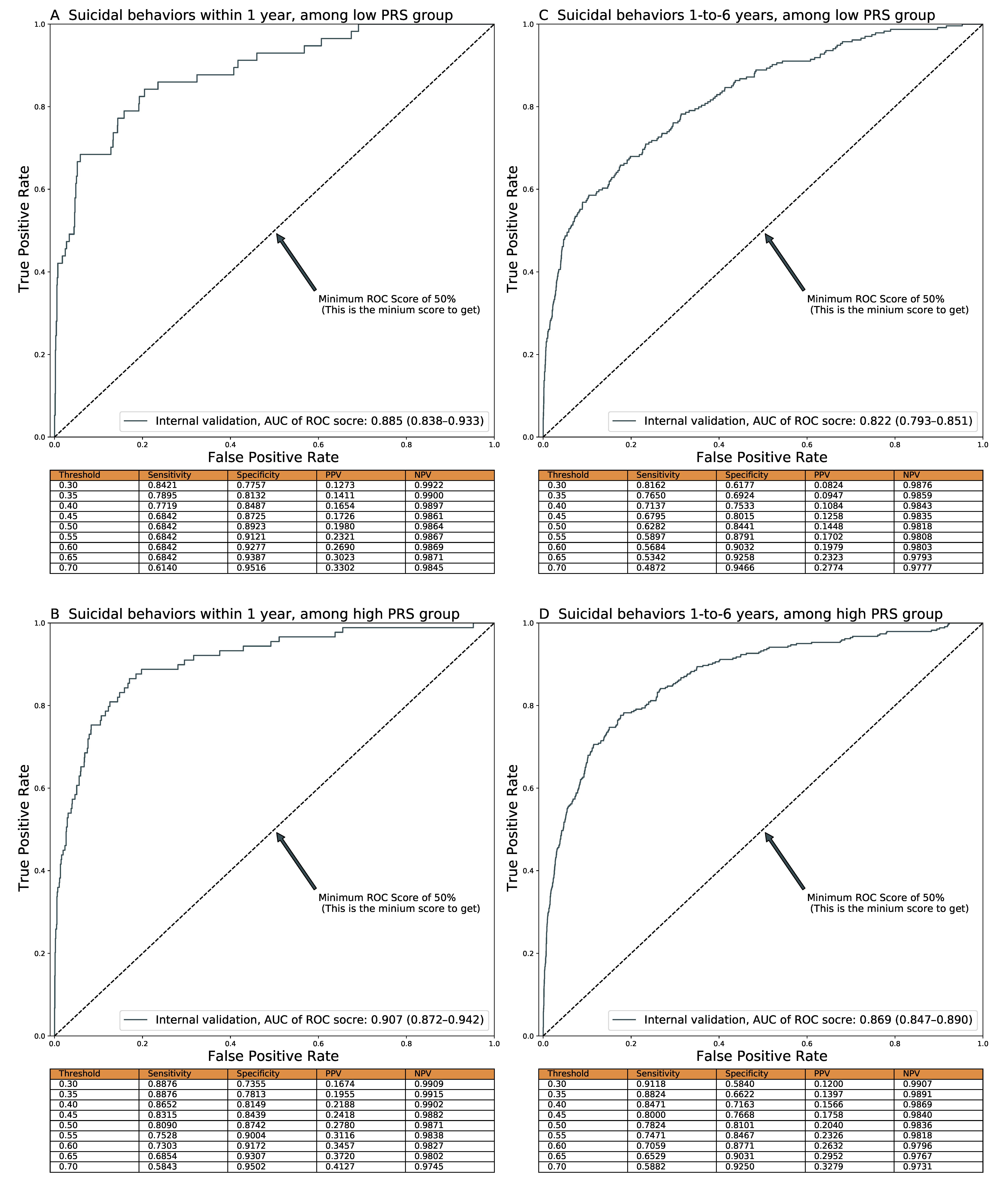** |
| --- |
| Figure S5 The performance of applicable models, based on top 20 features identified by the full prediction models, among individuals with low and high genetic susceptibilities to suicidality  The area under the receiver operator curve (AUC-ROC).  The above tables showed the internal validation performance (sensitivity, specificity, positive predictive value (PPV), and negative predictive value (NPV)) of suicide predictions models at different classified threshold. |

| **Table S1: The loadings of PCA.** | | | |
| --- | --- | --- | --- |
|  | Western | Prudent | Open sandwich |
| Cooked vegetable intake | 0.015 | 0.546 | 0.120 |
| Salad raw vegetable intake | -0.090 | 0.599 | 0.034 |
| Fresh fruit intake | -0.108 | 0.537 | -0.189 |
| Dried fruit intake | -0.112 | 0.406 | -0.031 |
| Oily fish intake | 0.134 | 0.593 | -0.017 |
| Nonoily fish intake | 0.223 | 0.494 | 0.068 |
| Processed meat intake | 0.527 | -0.187 | 0.391 |
| Poultry intake | 0.566 | 0.093 | -0.151 |
| Beef intake | 0.710 | -0.086 | 0.003 |
| Lamb mutton intake | 0.712 | 0.035 | -0.004 |
| Pork intake | 0.704 | -0.010 | 0.055 |
| Cereal intake | -0.072 | 0.197 | -0.177 |
| Bread intake | 0.005 | -0.043 | 0.694 |
| Cheese intake | -0.073 | 0.030 | 0.498 |
| Water intake | -0.034 | 0.347 | -0.308 |
| Tea intake | -0.011 | 0.097 | 0.431 |
| Hot drink temperature | -0.026 | -0.046 | 0.037 |
| Salt added to food | 0.121 | -0.183 | 0.226 |

| **Table S2: The coding book.** | | | |
| --- | --- | --- | --- |
| **Columns** | **Type of features** | **Feature processing Method** | **Source** |
| PRS | polytomous | The polygenic risk scores (PRS) were computed by LDPred2 and were divided into “low risk” (the lowest third of PRS), “intermediate risk” (second third), “high risk” (highest third). | Genomics data |
| Whether had a pace-maker before the body impedance measures | binary |  | Field-3079 |
| Waist circumference | numeric |  | Field-48 |
| Hip circumference | numeric |  | Field-49 |
| Whether had drank caffeine within the hour prior to doing the spirometry test | binary |  | Field-3089 |
| Body mass index | numeric |  | Field-21001 |
| Body fat percentage | numeric |  | Field-23099 |
| How many years have you lived at your current address? | numeric | -10 to 0.5; -1 and -3 to 'Missing'; | Field-699 |
| How many people are living together in your household? | numeric | -1 and -3 to 'Missing'; | Field-709 |
| Average total household income before tax | polytomous | -1 and -3 to 'Missing'; | Field-738 |
| In a typical week, on how many days did you walk for at least 10 minutes at a time? | numeric | -2 to 0; -1 and -3 to 'Missing'; | Field-864 |
| How many minutes did you usually spend walking on a typical day? | numeric | -1 and -3 to 'Missing'; | Field-874 |
| Number of days of moderate physical_activity_10_minutes in a typical week | numeric | -1 and -3 to 'Missing'; | Field-884 |
| In a typical week, how many days did you do 10 minutes or more of vigorous physical activity? | numeric | -1 and -3 to 'Missing'; | Field-904 |
| How would you describe your usual walking pace? | polytomous | -1 and -3 to 'Missing'; | Field-924 |
| At home, during the last 4 weeks, about how many times a day do you climb stairs? | polytomous | -1 and -3 to 'Missing'; | Field-943 |
| In a typical day in summer, how many hours do you spend outdoors? | numeric | -10 to 0.5; -1 and -3 to 'Missing'; | Field-1050 |
| In a typical day in winter, how many hours do you spend outdoors? | numeric | -10 to 0.5; -1 and -3 to 'Missing'; | Field-1060 |
| In a typical day, how many hours do you spend watching TV? | numeric | -10 to 0.5; -1 and -3 to 'Missing'; | Field-1070 |
| In a typical day, how many hours do you spend using the computer? | numeric | -10 to 0.5; -1 and -3 to 'Missing'; | Field-1080 |
| In a typical day, how many hours do you spend driving? | numeric | -10 to 0.5; -1 and -3 to 'Missing'; | Field-1090 |
| How often do you drive faster than the speed limit on the motorway? | polytomous | -1 and -3 to 'Missing'; | Field-1100 |
| How many years of using a mobile phone at least once per week to make or receive calls? | character | -1 and -3 to 'Missing'; | Field-1110 |
| About how many hours sleep do you get in every 24 hours? (please include naps) | numeric | -1 and -3 to 'Missing'; | Field-1160 |
| On an average day, how easy do you find getting up in the morning? | polytomous | -1 and -3 to 'Missing'; | Field-1170 |
| Do you consider yourself to be morning/evening person? | polytomous | -1 and -3 to 'Missing'; | Field-1180 |
| Do you have a nap during the day? | polytomous | -3 to 'Missing'; | Field-1190 |
| Do you have trouble falling asleep at night or do you wake up in the middle of the night? | polytomous | -3 to 'Missing'; | Field-1200 |
| Does your partner or a close relative or friend complain about your snoring? | binary | -1 and -3 to 'Missing'; | Field-1210 |
| How likely are you to doze off or fall asleep during the daytime when you don't mean to? | polytomous | -1 and -3 to 'Missing'; | Field-1220 |
| Do you smoke tobacco now? | polytomous | -3 to 'Missing'; | Field-1239 |
| In the past, how often have you smoked tobacco? | polytomous | -3 to 'Missing'; | Field-1249 |
| Does anyone in your household smoke? | polytomous | -3 to 'Missing'; | Field-1259 |
| At home, about how many hours per week are you exposed to other people's tobacco smoke? | numeric | -1 and -3 to 'Missing'; | Field-1269 |
| Does your diet vary much from week to week? | polytomous | -1 and -3 to 'Missing'; | Field-1548 |
| About how often do you drink alcohol? | polytomous | -3 to 'Missing'; | Field-1558 |
| Compared to 10 years ago, do you drink? | polytomous | -1 and -3 to 'Missing'; | Field-1628 |
| Comparative body size at age 10 | polytomous | -1 and -3 to 'Missing'; | Field-1687 |
| Comparative height size at age 10 | polytomous | -1 and -3 to 'Missing'; | Field-1697 |
| What best describes the colour of your skin without tanning? | polytomous | -1 and -3 to 'Missing'; | Field-1717 |
| What would happen to your skin if it was exposed to sunlight without protection? | polytomous | -1 and -3 to 'Missing'; | Field-1727 |
| Do people say about your facial ageing | polytomous | -1 and -3 to 'Missing'; | Field-1757 |
| Were you adopted as a child? | binary | -1 and -3 to 'Missing'; | Field-1767 |
| Are you a twin, triplet or other multiple birth? | binary | -1 and -3 to 'Missing'; | Field-1777 |
| Did your mother smoke regularly around the time when you were born? | binary | -1 and -3 to 'Missing'; | Field-1787 |
| Is your father still alive? | binary | -1 and -3 to 'Missing'; | Field-1797 |
| Is your mother still alive? | binary | -1 and -3 to 'Missing'; | Field-1835 |
| How many brothers do you have? | numeric | -1 and -3 to 'Missing'; | Field-1873 |
| How many sisters do you have? | numeric | -1 and -3 to 'Missing'; | Field-1883 |
| Does your mood often go up and down? | binary | -1 and -3 to 'Missing'; | Field-1920 |
| Do you ever feel 'just miserable' for no reason? | binary | -1 and -3 to 'Missing'; | Field-1930 |
| Are you an irritable person? | binary | -1 and -3 to 'Missing'; | Field-1940 |
| Are your feelings easily hurt? | binary | -1 and -3 to 'Missing'; | Field-1950 |
| Do you often feel 'fed-up'? | binary | -1 and -3 to 'Missing'; | Field-1960 |
| Would you call yourself a nervous person? | binary | -1 and -3 to 'Missing'; | Field-1970 |
| Are you a worrier? | binary | -1 and -3 to 'Missing'; | Field-1980 |
| Would you call yourself tense or 'highly strung'? | binary | -1 and -3 to 'Missing'; | Field-1990 |
| Do you worry too long after an embarrassing experience? | binary | -1 and -3 to 'Missing'; | Field-2000 |
| Do you suffer from 'nerves'? | binary | -1 and -3 to 'Missing'; | Field-2010 |
| Do you often feel lonely? | binary | -1 and -3 to 'Missing'; | Field-2020 |
| Are you often troubled by feelings of guilt? | binary | -1 and -3 to 'Missing'; | Field-2030 |
| Would you describe yourself as someone who takes risks? | binary | -1 and -3 to 'Missing'; | Field-2040 |
| Over the past two weeks, how often have you felt down, depressed or hopeless? | polytomous | -1 and -3 to 'Missing'; | Field-2050 |
| Over the past two weeks, how often have you had little interest or pleasure in doing things? | polytomous | -1 and -3 to 'Missing'; | Field-2060 |
| Over the past two weeks, how often have you felt tense, fidgety or restless? | polytomous | -1 and -3 to 'Missing'; | Field-2070 |
| Over the past two weeks, how often have you felt tired or had little energy? | polytomous | -1 and -3 to 'Missing'; | Field-2080 |
| Have you ever seen a general practitioner for nerves, anxiety, tension or depression? | binary | -1 and -3 to 'Missing'; | Field-2090 |
| Have you ever seen a psychiatrist for nerves, anxiety, tension or depression? | binary | -1 and -3 to 'Missing'; | Field-2100 |
| How often are you able to confide in someone close to you? | polytomous | -1 and -3 to 'Missing'; | Field-2110 |
| Age first had sexual intercourse | numeric | -2 to 0; -1 and -3 to 'Missing'; | Field-2139 |
| Have you ever had sexual intercourse with someone of the same sex? | binary | -3 to 'Missing'; | Field-2159 |
| Do you have any long-standing illness, disability or infirmity? | binary | -1 and -3 to 'Missing'; | Field-2188 |
| Do you wear glasses or contact lenses to correct your vision? | binary | -3 to 'Missing'; | Field-2207 |
| Age started wearing glasses or contact lenses | numeric | -1 and -3 to 'Missing'; | Field-2217 |
| Do you have any other problems with your eyes or eyesight? | polytomous | -3 to 'Missing'; | Field-2227 |
| Do you play computer games? | polytomous | -3 to 'Missing'; | Field-2237 |
| Do you wear sun protection when you spend time outdoors in the summer? | polytomous | -3 to 'Missing'; | Field-2267 |
| How many times a year would you use a solarium or sunlamp? | numeric | -10 to 0.5; -1 and -3 to 'Missing'; | Field-2277 |
| In the last year have you ever had wheeze or whistling in the chest? | binary | -1 and -3 to 'Missing'; | Field-2316 |
| Do you ever have any pain or discomfort in your chest? | binary | -1 and -3 to 'Missing'; | Field-2335 |
| Have you fractured/broken any bones in the last 5 years? | binary | -1 and -3 to 'Missing'; | Field-2463 |
| Current employment status | polytomous | -7 and -3 to 'Missing'; | Field-6142 |
| Current/past smoking status | polytomous | -3 to 'Missing'; | Field-20116 |
| Current/past alcohol drinker status | polytomous | -3 to 'Missing'; | Field-20117 |
| Gender | binary |  | Field-31 |
| Index of Multiple Deprivation | character | The scores of indices of multiple deprivations were divided into “low” (the lowest third), “intermediate ” (second third), “high” (highest third). | Field-26410, 26426, 26427 |
| Myocardial_infarction_1 | binary | hospitalization with any corresponding diagnosis within 1 year before recruited assessment. | HESIN and HESIN_DIAG |
| Congestive_heart_failure_1 | binary |  |  |
| Peripheral_vascular_disease_1 | binary |  |  |
| Cerebrovascular_disease_1 | binary |  |  |
| Dementia_1 | binary |  |  |
| Chronic_pulmonary_disease_1 | binary |  |  |
| Connective_tissue_disease_1 | binary |  |  |
| Ulcer_disease_1 | binary |  |  |
| Mild_liver_disease_1 | binary |  |  |
| Diabetes_mellitus_1 | binary |  |  |
| Hemiplegia_1 | binary |  |  |
| Moderate_or_severe_renal_disease_1 | binary |  |  |
| Diabetes_mellitus_with_chronic_complications_1 | binary |  |  |
| Any_tumor_1 | binary |  |  |
| Leukemia_1 | binary |  |  |
| Lymphoma_1 | binary |  |  |
| Moderate_or_severe_liver_disease_1 | binary |  |  |
| Metastatic_solid_tumor_1 | binary |  |  |
| AIDS_1 | binary |  |  |
| Myocardial_infarction_4 | binary | hospitalization with any corresponding diagnosis during 1-to-4 years before recruited assessment. | HESIN and HESIN_DIAG |
| Congestive_heart_failure_4 | binary |  |  |
| Peripheral_vascular_disease_4 | binary |  |  |
| Cerebrovascular_disease_4 | binary |  |  |
| Dementia_4 | binary |  |  |
| Chronic_pulmonary_disease_4 | binary |  |  |
| Connective_tissue_disease_4 | binary |  |  |
| Ulcer_disease_4 | binary |  |  |
| Mild_liver_disease_4 | binary |  |  |
| Diabetes_mellitus_4 | binary |  |  |
| Hemiplegia_4 | binary |  |  |
| Moderate_or_severe_renal_disease_4 | binary |  |  |
| Diabetes_mellitus_with_chronic_complications_4 | binary |  |  |
| Any_tumor_4 | binary |  |  |
| Leukemia_4 | binary |  |  |
| Lymphoma_4 | binary |  |  |
| Moderate_or_severe_liver_disease_4 | binary |  |  |
| Metastatic_solid_tumor_4 | binary |  |  |
| AIDS_4 | binary |  |  |
| Handgrip strength | numeric | Averaging the multiple instance results. | Field-46, 47 |
| Pulse rate | numeric |  | Field-102 |
| Diastolic blood pressure | numeric |  | Field-4079 |
| Systolic blood pressure | numeric |  | Field-4080 |
| Western dietary pattern | polytomous | The scores of this dietary pattern were calculated through principal component analysis (PCA) of standardized diet questionnaires. And then we divided the dietary pattern scores into three levels by descending rank: high, middle, and low. | Field-1289, 1299, 1309, 1319, 1339, 1349, 1359, 1369, 1379, 1389, 1408, 1438, 14581 1478, 1488, 1518, 1528, and 6144; |
| Prudent dietary pattern | polytomous |  |  |
| Open sandwich dietary pattern | polytomous |  |  |
| Whether own accommodation lived in? | binary | -7 and -3 to 'Missing'; 1 to 'Yes'; 2~6 to 'No'; | Field-680 |
| Have you made any major changes to your diet in the last 5 years? | binary | -3 to 'Missing'; 1~2 to 'Yes'; 0 to 'No'; | Field-1538 |
| Whether born not in uk? | binary | -7 and -3 to 'Missing'; 6 to 'Yes'; 1~5 to 'No'; | Field-1647 |
| Are you right or left handed? | polytomous | -3 to 'Missing'; | Field-1707 |
| Compared with one year ago, has your weight changed? | polytomous | -1 and -3 to 'Missing'; | Field-2306 |
| In the last year have you had any falls? | polytomous | -3 to 'Missing'; | Field-2296 |
| In general how would you rate your overall health? | polytomous | -1 and -3 to 'Missing'; | Field-2178 |
| Do you have any difficulty with your hearing? | polytomous | -1 and -3 to 'Missing'; | Field-2247 |
| History of psychiatric disorders | binary |  | HESIN，HESIN_DIAG, and Primary care |
| History of suicide attempt | binary |  | HESIN，HESIN_DIAG |
| Age | numeric |  | Field-21003 |
